# Hump-nosed viper bite: an important but under-recognized cause of systemic envenoming

**DOI:** 10.1186/1678-9199-20-24

**Published:** 2014-06-06

**Authors:** Mitrakrishnan Chrishan Shivanthan, Jevon Yudhishdran, Rayno Navinan, Senaka Rajapakse

**Affiliations:** 1 University Medical Unit, National Hospital of Sri Lanka, Colombo, Sri Lanka; 2 Medical Unit, National Hospital of Sri Lanka, Colombo, Sri Lanka; 3 Department of Clinical Medicine, and project lead, Tropical Medicine Research Unit, University of Colombo, Colombo, Sri Lanka

**Keywords:** *Hypnale*, Hump-nosed viper, Envenoming, Viper, Venom, Antivenom

## Abstract

Hump-nosed viper bites are common in the Indian subcontinent. In the past, hump-nosed vipers (*Hypnale* species) were considered moderately venomous snakes whose bites result mainly in local envenoming. However, a variety of severe local effects, hemostatic dysfunction, microangiopathic hemolysis, kidney injury and death have been reported following envenoming by *Hypnale* species. We systematically reviewed the medical literature on the epidemiology, toxin profile, diagnosis, and clinical, laboratory and postmortem features of hump-nosed viper envenoming, and highlight the need for development of an effective antivenom.

## Introduction

Hump-nosed viper (*Hypnale*) bite is an important yet under-recognized cause of morbidity and mortality in Southern India and Sri Lanka, where three species have been identified, namely *Hypnale hypnale*, *H. zara* and *H. nepa*. Specifically, *H. hypnale* has been reported from Sri Lanka and the Western Ghats of South India, while the other two species are endemic to Sri Lanka alone [1]. In Sri Lanka, this small pit viper is known as ‘polon-thelissa’ (viper with an upturned lip) or ‘kunakatuwa’ (referring to the necrotic effects at the bite site) in Sinhala, and ‘kopi viriyan’ (coffee snake) in Tamil. In Kerala, India, it is called ‘churrutta’ in the Malayalam language.

Although formerly thought to result only in minor or local envenoming, hump-nosed viper (HNV) bite is now known to cause serious systemic toxicity and fatalities [2-6]. Clinical manifestations of systemic envenoming by this snake include acute kidney injury, hematological manifestations, and other organ involvement, in some cases leading to death. Several unusual manifestations have also been reported. HNV is now recognized as a highly venomous snake, alongside the Indian cobra (*Naja naja*), the common krait (*Bungarus caeruleus*), Russell's viper (*Daboia russelii*) and the saw-scaled viper (*Echis carinatus*) [3]. It is the commonest cause of snakebite envenoming in Sri Lanka [7,8]. The clinical and epidemiological importance of HNV bite is under-recognized, and currently no effective antivenom exists. In this paper we review the published literature on the epidemiology, clinical manifestations, and treatment of envenoming following HNV bite.

We performed a systematic review of the published literature. The databases Medline, Embase and Scopus were searched using the terms ‘hypnale’ OR ‘hump nosed’ OR ‘hump-nosed viper’ in any field, with no date limits. The search was performed on March 22, 2014. A combined search of Medline and Embase resulted in 50 hits; Scopus produced 62 hits. Databases were merged and duplicates removed. The abstracts were read through by two authors independently (MCS and SR), and relevant papers identified. Related references were also included. Data was extracted by two authors (MCS and SR), and crosschecked by the other two authors (MRN and MJY). Where conflicting data were present, a consensus was arrived upon by discussion between all the authors. The inter-rater agreement was 100%. The full text was obtained in 45 papers. A total of 40 papers provided useful data.

## Review

### Epidemiology

HNV accounts for between 27 and 77% of venomous snakebites in Sri Lanka and south India [4,5,8,9]. In southwestern India (Kerala), in the year 2007, *H. hypnale* was identified as a common and dangerous source of envenoming, second only to Russell's viper. Deaths have been subsequently recorded in India following HNV envenoming [5,10]. In a prospective multicenter study involving ten hospitals in Sri Lanka, 302 HNV bite victims were identified out of 860 patients with bites by identified snakes (35%). These were mostly men between the ages 11 and 51 years, bitten commonly at night on their feet or ankles near their homes [4]. The case fatality rate resulting from HNV bite was 1.7%, all due to acute kidney injury. Similar findings had been reported previously in a single-center prospective study in Avissawella, Sri Lanka, although fatalities were not seen [11].

HNV was the commonest amongst cases of identified snakebite, in a three-year hospital based observational study from 2006 in the central hills of Sri Lanka [12]. Notably, the majority (61%) of victims had been bitten in a home garden. However, in this study there were large numbers of unidentified snakebites that did not result in envenoming. Another recent prospective hospital-based clinical study in Sri Lanka of proven HNV bites identified 114 definite cases [2]. Most bites were on the lower limbs and during the daytime. *H. hypnale* bites were usually in home gardens, whilst *H. zara* and *H. nepa* bites occurred in the forests and tea estates respectively. In a large case series spanning 1990–2008, of 1543 patients with HNV bite, 4.34% were found to have developed systemic envenoming; only two patients died [13]. The pattern of bites among children may differ slightly from that of the adult population. One study in the pediatric population showed that bites in children occur most commonly on the feet, and then the hands, mostly following provocation, and take place between 5 and 10 pm [14]. Overall, systemic envenoming is seen in a significant proportion of patients with HNV bite, although mortality appears to be low. Most reported cases of systemic envenoming are with *H. hypnale*[2]. A single case report describes coagulopathy and acute kidney injury leading to death after confirmed *H. zara* envenoming [15]. There are no reported cases of systemic envenoming or fatality due to a *H. nepa* bite.

### Toxin profile

*In vitro* studies have found that the three *Hypnale* venoms in Sri Lanka display similar chromatographic profiles [16]. Potent cytotoxicity, weak neurotoxic and myotoxic activity, mild procoagulant activity and also phospholipase activity are present in HNV venom. These activities are not neutralized by the locally available polyvalent antivenom. High activity levels of thrombin-like enzyme, proteases, phospholipase A_2_, L-amino acid oxidase and hyaluronidase may explain the hematological manifestations described below. The thrombin-like enzyme, which is highly acidic, is likely to exist in multiple isoforms (as evidenced by its chromatographic patterns). The hemorrhagic and necrotic activities of the venom are probably associated with the proteolytic enzyme found mainly in the basic fraction [17]. *In vivo* studies in mice also suggest that *H. hypnale* venom is more toxic compared to the other two species, with histopathological changes as well as lethality occurring with lower doses of venom [18].

### Diagnosis

Identification of the snake by medical personnel (or sometimes a herpetologist) viewing the dead snake or a photograph is the commonest means of diagnosing *Hypnale* snakebite [2,9,13]. However, misidentification rates as high as 6% by hospital staff have been reported [19]. Misidentification commonly occurs between Russell’s viper and the saw-scaled viper, which may contribute to confusion regarding clinical manifestations of severity [5,19]. Where the snake is unidentified, syndromic diagnosis is a very useful tool; this is frequently used in clinical settings [19]. The basis of syndromic diagnosis is that, where the snake has not been identified visually, it is possible to make an identification based on the clinical syndrome it produces, using a validated algorithm. Its specificity for Russell’s viper and HNV is high, albeit with poor sensitivity. The key clinical features in this tool favoring HNV bite are local envenoming, incoagulable blood, and renal failure; however, these are also provoked by Russell’s viper [19].

Alternate scoring systems have been suggested but are insensitive in diagnosing HNV and saw scaled viper envenoming, although they help differentiate HNV bite from Russell's viper, krait and cobra bites [20]. Immune assays based on the antigenic profile of viper toxins that may differentiate between species of vipers have been tested. These have the potential to become valuable tools in diagnosing and differentiating HNV bite from other snakebites [21]. Cross-reactivity studies using indirect ELISA have shown that anti-*Hypnale hypnale* IgG cross-reacts extensively with several Asiatic crotalid venoms. Further studies have shown that this assay is able to distinguish and quantify venoms of *H. hypnale, Daboia russelii* and *Echis carinatus sinhaleyus.*

### Envenoming

#### ***Local effects***

Pain and fang marks are invariably found after HNV bite. Local envenoming is the commonest manifestation, with an incidence of approximately 90% [2,4]. This can be severe, and lead to tissue necrosis. Local pain, swelling, induration, local hemorrhagic blister formation, local bleeding, local necrosis, need for amputation and skin grafting, and regional lymphadenopathy have all been reported [2,4,11,22]. However, severe local envenoming with proximal spread is relatively less common, and was seen in only 16 of 114 study subjects in a recent hospital-based study [2].

### ***Systemic envenoming***

Although HNV bite was previously thought only to result in local envenoming, systemic effects are now a recognized phenomenon, with more than one organ system being affected [2,4,11,13]. The variable frequency and severity of systemic envenoming could at least partly be explained by variations in toxicity between the different *Hypnale* species [2]. HNV bites appear to result in a higher incidence of both local and systemic effects in children, especially those under three years; systemic effects appear to occur early on, and show an inverse relationship to body surface area [14].

### ***Vascular and hematological manifestations***

Individual reports of coagulopathy following hump-nosed viper envenoming were reported in the 1990s [23-25]. In a prospective study of 56 patients with proven HNV bites, 12 patients (21.4%) developed continued oozing of blood from the site of the bite, with prolonged clotting time [24]. Low fibrinogen levels and increased fibrinogen degradation products in plasma were also demonstrated in these patients. Bleeding time, platelet count, prothrombin time, and partial thromboplastin time with kaolin were normal. The authors suggested the use of whole-blood clotting time to monitor cases of hump-nosed viper bite; this is standard practice in hospitals in Sri Lanka now. In a recent paper on syndromic diagnosis of snakebite, the authors suggested that coagulopathy was present in 39% of 302 patients with HNV bite, making it the second commonest manifestation after local envenoming [19]. Even where the whole-blood clotting time is normal, mild coagulopathy, evidenced by slight elevation of INR, low fibrinogen and factors V and VIII have been associated with HNV bite [26]. It is postulated that these abnormalities occur due to a thrombin-like enzyme contained in the venom; the clinical significance of these abnormalities is unclear.

Microangiopathic hemolytic anemia with thrombocytopenia, coagulopathy, fibrinolysis, thrombocytopenia or spontaneous systemic hemorrhage, and a case of hemolytic uremic syndrome, have been reported with HNV bite [5,6,26-28]. Consumptive coagulopathy has been purported to be the mechanism for these manifestations in some cases [27,28]. Isbister [29] described this venom-induced coagulopathy as constituting the commonest coagulopathy associated with snake envenoming. This has been likened to disseminated intravascular coagulation (DIC), because of certain similarities seen between the two conditions, e.g., elevated D-dimer, prolonged prothrombin time, and low fibrinogen; however, important differences between venom-induced coagulopathy and DIC have been demonstrated [29]. The onset and resolution were rapid, while there was no evidence of either systemic microthrombi or end-organ failure. In a portion of patients with venom-induced coagulopathy, however, a clinical syndrome consistent with thrombotic microangiopathy has been reported, characterized by thrombocytopenia, microangiopathic hemolytic anemia and acute kidney injury. This venom induced coagulopathy-related thrombotic microangiopathy appears to occur with several different snakebites worldwide, including vipers and elapids.

### ***Renal toxicity***

In mouse model studies, histopathogical examination of the kidneys have revealed proximal tubular cell injury and acute tubular necrosis with intact basement membrane, indicating possible direct nephrotoxicity following HNV envenoming [18]. Renal toxicity was observed in around 19% of cases with HNV bite in a recent study, while death following acute kidney injury is well documented [4,6,30,31]. Renal toxicity is characterized by features of acute kidney injury, i.e., oliguria, dark urine, fluid retention, uremia and elevated serum creatinine, often requiring dialysis [4-6,14,15,23,27,28,30,32]. Both enzymatic activity in venom and immunologic mechanisms have been implicated in causing renal damage following snakebite, with the latter playing only a minor role. Hemodynamic alterations caused by vasoactive mediators and cytokines also contribute to renal damage [33]. Bilateral cortical necrosis, characterized by irregular areas of coagulative necrosis involving both glomeruli and tubules in the cortices, have been demonstrated in postmortem studies as far back as in 1970 [30]. Similar postmortem findings have been described in a case of *H. zara* envenoming complicated by coagulopathy [15].

In a prospective observational study carried out at the Nephrology Unit, Kandy, Sri Lanka from October 2010 to October 2011, out of 11 patients with acute kidney injury following HNV bites, all required renal replacement therapy, seven developed thrombotic microangiopathy with evidence of microangiopathic hemolytic anemia (MAHA), thrombocytopenia and severe anemia necessitating multiple blood transfusions, and two progressed to chronic renal failure [6]. Autopsy in two fatalities demonstrated thrombotic microangiopathy. The authors queried a causal link between thrombosis and renal damage. Chronic kidney disease, whose occurrence is predicted by the duration of renal replacement therapy and failure to normalize renal function after one year, is also known to occur following HNV bite. Glomerulosclerosis is the predominant histological finding [34,35].

### ***Neurological effects***

Direct neurological toxicity or effects on the neuromuscular junction have not been described in relation to HNV envenoming. EEG abnormalities without clinical neurological derangement have been observed in humans following HNV envenoming [22]. There is a single case report of an ischemic stroke following HNV bite. In this instance, the platelets were normal, and there was no evidence of coagulopathy, although acute kidney injury that had been present was resolved. It was suggested that the pathophysiology was of a vaso-occlusive nature [32].

### ***Effects on the heart***

Transient ECG changes and symptoms suggestive of cardiac involvement have been observed with HNV envenoming. Electrolyte imbalance and hemodynamic instability could contribute towards these. However, no evidence of myocardial damage, characterized by elevated cardiac troponin, has been demonstrated with HNV envenoming [36].

### ***Pulmonary effects***

Pulmonary edema has been documented in humans following envenoming by *Hypnale hypnale* or *Hypnale zara*[5,15]. However, there is no conclusive evidence to suggest that lung injury occurs as a specific effect of HNV envenoming. Studies in mice have shown that HNV venom can induce pulmonary hemorrhage [18].

### ***Gastrointestinal and liver effects***

Nausea, vomiting, hematemesis (probably secondary to coagulopathy) and abdominal pain have been reported [4]. However, hepatic dysfunction has not been described.

### Treatment

Treatment is mainly symptomatic and supportive. Analgesics, splinting of the bitten limb, wound care, and surgical management including skin grafting constitute the common approach employed against local envenoming. In rare cases, amputation may be needed. Antibiotics are often used routinely, despite lack of benefit in snakebite, and tetanus toxoid is also administered [4,14,37]. Features of systemic envenoming are treated with supportive care. Renal replacement therapy is the mainstay of management of acute renal impairment, and may become necessary if chronic kidney disease occurs consequently [6,34,35]. Optimized administration of blood components, correction of coagulopathy, and plasma exchange may be required when hematologic derangements occur. No clear trial based evidence is available showing benefit of these interventions. There are no specific protocols for managing envenoming following HNV bite, and standard guidelines for snakebite management are followed [38].

### Antivenom

No specific or polyvalent antivenom is available yet for human use in HNV envenoming [39]. The currently used polyvalent antivenom is ineffective in the treatment of HNV envenomation, and is associated with a high incidence of reactions [4,9,40]. *In vivo* animal studies evaluating potential alternative antivenom preparations for HNV show mixed results [21]. Trials in mice have found hematopolyvalent antivenom to be efficacious following experimental envenoming with *H. hypnale* venom. This was shown to effectively prevent acute kidney injury and death [41]. *In vitro* neutralization studies showed that the hematopolyvalent antivenom effectively neutralized the lethality of *H. hypnale* venom, as well as the hemorrhagic, procoagulant, nephrotoxic, and necrotic activities of the venom [39]. The monovalent *Calloselasma rhodostoma* antivenom also is also capable of neutralizing the lethality and toxic activities of the venom, but its potency was lower [41]. *H. hypnale* venom elicited satisfactory titers of anti-*Hypnale hypnale* IgG in rabbits after the third immunization. This anti-*Hypnale hypnale* IgG, isolated using the caprylic acid precipitation method, was effective in neutralizing venom lethality (potency = 48 LD_50_ per mL IgG) as well as its procoagulant, hemorrhagic and necrotic effects. This suggests the potential to produce a specific antivenom [21].

### Outcome

Survival and minor effects is the norm after HNV bites, although fatalities are known to occur [2,4,11]. Chronic kidney disease is a recognized complication, as described above.

## Conclusions

Formerly thought to result only in local envenoming, HNV bite can result in serious sequelae including death. Acute kidney injury and hematological manifestations are the predominant serious effects. Clinically significant neurotoxicity or cardiotoxicity does not occur. There are two key areas for future research: firstly, identification of clinical parameters and biochemical tests for early identification of systemic envenoming, i.e., early detection of coagulopathy and nephrotoxicity; secondly, the development of an efficacious specific antivenom for routine use.

## Competing interests

The authors declare that there are no competing interests.

## Authors' information

MCS and MJY are senior specialist registrars, and MRN is a specialist registrar at the National Hospital, Colombo, Sri Lanka. SR is a professor and consultant physician, Department of Clinical Medicine, and project lead, Tropical Medicine Research Unit, University of Colombo, Sri Lanka.

## Authors’ contributions

MCS and SR conceived the review. MCS, MJY and MRN performed the initial literature search. MCS and SR independently read through the abstracts, selected relevant papers, and read through the full texts. MCS, MJY and SR extracted data. MCS wrote the first draft. SR and MRN critically reviewed and revised the manuscript. All authors read and approved the final manuscript.
